# Mechanisms of lipid malabsorption in Cystic Fibrosis: the impact of essential fatty acids deficiency

**DOI:** 10.1186/1743-7075-2-11

**Published:** 2005-05-03

**Authors:** N Peretti, V Marcil, E Drouin, E Levy

**Affiliations:** 1 Department of Nutrition, CHU-Sainte-Justine, Université de Montréal, Montréal, Québec, Canada; 2 Department of Pediatrics, CHU-Sainte-Justine, Université de Montréal, Montréal, Québec, Canada

## Abstract

Transport mechanisms, whereby alimentary lipids are digested and packaged into small emulsion particles that enter intestinal cells to be translocated to the plasma in the form of chylomicrons, are impaired in cystic fibrosis. The purpose of this paper is to focus on defects that are related to intraluminal and intracellular events in this life-limiting genetic disorder. Specific evidence is presented to highlight the relationship between fat malabsorption and essential fatty acid deficiency commonly found in patients with cystic fibrosis that are often related to the genotype. Given the interdependency of pulmonary disease, pancreatic insufficiency and nutritional status, greater attention should be paid to the optimal correction of fat malabsorption and essential fatty acid deficiency in order to improve the quality of life and extend the life span of patients with cystic fibrosis.

## I Introduction

Cystic fibrosis (CF) is the most common autosomal recessive genetic disease observed in the Caucasian population, affecting about 1 in 2,500 newborns. It is caused by mutations in the Cystic Fibrosis Transmembrane Conductance Regulator (CFTR) gene discovered in 1989. The gene was cloned from chromosome 7q21-31, is composed of 27 exons and encodes a protein that functions as a chloride channel [1-4]. CFTR has 1,480 amino acids and the molecular weight varies from 140 kDA to 170 kDA, depending upon the degree of posttranslational glycation in the Golgi complex, which appears to vary somewhat according to cell type and genotype. Defective cAMP-dependent chloride ion conductance leads to an imbalance in fluid flow across epithelia, thickened mucus and blocked exocrine ducts in the affected tissues. In addition to respiratory-tract manifestations that represent the major cause of death in CF, the vast majority of young CF subjects have a wide variability of clinical expression, including gastrointestinal, metabolic and nutritional problems [5,6]. Advances in medical management have led to a continual improvement of life expectancy for CF patients. However, intestinal fat malabsorption remains a persistent feature, given the difficulty of achieving complete correction in clinical practice despite the remarkable benefits resulting from exogenous pancreatic enzyme replacement therapy. [7-11]. The purpose of this article is to present a systematic critical review of literature and data sources pertaining to fat malabsorption in CF, emphasizing the role of abnormal, intraluminal and intracellular factors. Furthermore, the relationship between essential fatty acid deficiency (EFAD) and intestinal fat transport is particularly underscored given its implication in gastrointestinal pathophysiology. To facilitate the reader's appreciation of major gastroenterological problems in CF, we first describe the normal digestive and absorptive processes before addressing the issue of various defective mechanisms in CF.

## II Lipids and Essential Fatty Acids (EFAs)

Dietary fat intake represents 35% (100 to 150 g per day) of total calories consumed in the North American diet [12]. Dietary fats are composed mainly of triacylglycerol (TG) with 92–96% long-chain fatty acids [13]. In addition to dietary intake, lipids are derived from bile and desquamated mucosal cells.

Lipids have varied as well as essential functions in organisms. TGs are the principal reserve supply of energy, while phospholipids (PLs) and cholesterol are crucial for the elaboration of cell membranes, and play a functional role in steroid hormone and biliary acid (BA) synthesis. Linoleic (18:2n-6) and linolenic (18:3n-3) acids have recognized functions in growth, the development of the central nervous system, immune and vascular functions, eicosanoid production, membrane fluidity and the control of lipid homeostasis. Adequate EFA levels depend entirely on adequate dietary intake and absorption because mammalian cells cannot synthesize *de novo *EFA efficiently. Once these EFAs are appropriately absorbed, they can be converted into long-chain polyunsaturated fatty acids (PUFAs) such as arachidonic acid (AA, 20:4n-6), eicosapentaenoic (EPA, 20:5n-3) and docosahexaenoic acid (DHA, 22:6n-3). Conditions leading to fat malabsorption, as in CF, have been associated with a high incidence of EFAD. However, diet-induced EFAD impairs dietary lipid absorption through various mechanisms, which will be thoroughly discussed sin this review.

### II.1. CF Patients and EFAD

A high EFAD incidence (85%) has been frequently reported in CF patients [14-19]. EFAD is most marked at infancy [20] and EFA impairment has been observed most significantly in the cholesteryl ester (CE) fraction [15,21]. The most often reported abnormalities in plasma are a decrease in linoleic acid and AA, its major metabolites, as well as DHA [22]. This EFA diminution is associated with an elevation in eicosatrienoic acid (20:3n-9), oleic acid (18:1n-9) and palmitoleic acid (16:1n-7) [14,23,24], resulting in a rise in the triene/tetraene ratio. It has been suggested that the (20:3n-9/20:4n-6) ratio is a very sensitive and reliable index of EFAD. A value of the ratio above 0.2 indicates an appropriate cutoff for the diagnosis of EFAD [25]. However, results obtained from CF studies vary, possibly as a consequence of different nutritional status, the degree of malabsorption and the severity of the CFTR mutation.

Abnormal EFA status in CF patients does not seem to be the consequence of intestinal malabsorption only, since reduced EFA values are found as early as in the first weeks of life in infants with CF [22] and they are also present in well-nourished young CF patients who do not receive a low-fat diet and do not present with fat malabsorption [17,24,26-28] even with regular pancreatic enzyme therapy [21]. Among other mechanisms related to EFAD, one can mention the excessive oxidation of EFA as an energy source [29], the exaggerated utilization of eicosanoids as precursors of inflammatory response [30,31], the higher rate of lipid turnover in cell membranes [32], the impaired metabolism of EFA with a defect in plasma membrane incorporation, the decreased activity of desaturases [14,33] and lipid peroxidation [34].

### II.2. Relationship between CFTR and EFAD

CFTR primarily functions as a chloride channel in the apical membrane of the respiratory and gastro-intestinal tracts. An elevation of cAMP within the cell results in increased chloride transport through the CFTR. Movement of water is linked osmotically to such ion transport and chloride secretion represents one likely means of hydrating the mucosal surface of these organs. Other important studies have implicated the CFTR in additional functions, including the regulation of distinct Cl^- ^channel proteins, the modulation of intracellular vesicle and Golgi acidification, and the control of vesicle trafficking [35-40]. Furthermore, it has been suggested that the CFTR may regulate membrane PL composition. In human airway epithelial cultured cells, the incorporation of fatty acids (FAs) into membrane PLs is decreased when chloride conductance channels are chemically blocked [41]. A defective CFTR reduces the incorporation of linoleic acid in the PL of CF cells and transfecting these cells with the normal gene increases linoleic acid incorporation [42]. Thus, a relationship is possible between chloride transport conductance and EFA metabolism. To date, these studies are limited and additional investigation is needed to establish the role of the chloride channel function in EFA movement and metabolism.

CFTR knock-out mice demonstrate EFAD. But the EFA composition of the cell membrane varies with different animal models. In cftr^-/- ^mouse, the pancreas, lungs and ileum were deficient in (n-3) EFA [43], whereas in cftr ^tm1HGU/tm1HGU ^mice, the deficiency was in (n-6) EFA in plasma, lung and pancreatic PL [44]. The PUFA differences between the two mouse models emphasize the importance of determining the exact composition of membrane PL from healthy men and CF patients according to genotype in order to tailor appropriate EFA nutritional intervention.

In CF, AA levels suggest that abnormal EFA status may result from an impaired EFA metabolism. High levels of AA are also reported in the cell membrane of the ileum of CFTR knock-out mice [43]. The same AA imbalance has been reported in the PL fraction deriving from bronchial alveolar lavage fluid, plasma and tissues [31,45-48] of CF patients. The increased AA level in tissues may result from either overproduction or diminished catabolism. In CF cells (CFPAC-1), the conversion of 18:2n-6 into 20:4n-6 by desaturation and elongation processes is enhanced 1.8-fold compared with CF cells transfected with the normal gene [42]. AA overproduction is compatible with an intrinsic increase in AA release by CFTR-mutated respiratory epithelial cell lines [49]. A decreased metabolism of AA through cyclooxygenase, lipooxygenase or cytochrome P450 pathways could also explain high levels of AA in tissues, but this hypothesis should be thoroughly tested in CF because of a) the high rates of inflammatory species originating from AA that characterize CF patients; and b) the enhanced turnover of endogenous and exogenous AA in polymorph neutrophil granulocytes, which is possibly due to enhanced phospholipase A2 (PLA2) activity [50]. Then, AA catabolism is not obviously diminished, but the ratio between production and degradation may be unbalanced in favor of overproduction. Accordingly, it has been suggested that AA participates in CF physiopathology, since AA has been shown to inhibit CFTR Cl^- ^currents when applied to the cytoplasmic face of excised membrane patches [51,52]. Other FAs may also block CFTR pores with various levels of efficiency. For example, cis-unsaturated FAs are more effective than trans-unsaturated FAs and saturated FAs in interfering with CFTR function [52]. Furthermore, in respiratory epithelial cells, CFTR mutation ΔF508 is associated with an intrinsic increase in AA release [49] suggesting that the mechanism of chronic inflammation in CF, at least in part, involves this abnormality. Importantly, Chlore ion (Cl^-^) secretion across epithelial cells is induced by AA [53,54]. On the other hand, AA decreases Cl^- ^secretion across tracheal epithelium when its metabolism is impaired by cyclooxygenase and lipooxygenase inhibitors [54] suggesting that enhanced Cl^- ^secretion results from the action of its metabolites [52]. Furthermore, there is a correlation between EFAD and CF genotype in homozygotes for ΔF508 and heterozygotes for 394 delTT have displayed significantly lower concentrations of linoleic and DHA than the other mutations [55]. Thus, the relationship between AA metabolism abnormalities and specific mutations may explain the discrepancies observed with n-6 EFA supplementation [19,22,56-58]. Evidently, extensive studies are required to clarify the link between the CFTR and FAs, especially in view of the contribution of EFAD to CF pathophysiology.

In animals, EFAD induces symptoms similar to those in CF: defective Na^+ ^transport, increased bacterial colonization of airways, formation of clusters of lipid-laden macrophages in lungs [59], impaired alveolar macrophage function [60], alteration of surfactants [61], liver steatosis, decreased insulin secretion and increased caloric needs [62]. Interestingly, a membrane lipid imbalance plays a role in the phenotype expression in CFTR knock-out mice and feeding these animals with DHA has normalized disease-related changes that occur in epithelial cells and intestinal mucosa [63].

Of note is the finding that altered PL composition and structure can impair membrane fluidity [64-66], thereby modifying CFTR cell membrane localization and function. Membrane PL composition in CFTR knock-out mice is different from controls [44]. Apparently, lipid membrane composition regulates membrane protein activity through the association between lipid rafts and these proteins [67]. The transient residency of proteins in rafts emerges as a regulatory mechanism responsible for protein biological activity. Recently, it has been reported that lipid raft localization for CFTR is required for signaling in response to Pseudomonas aeruginosa infection, underlying the interaction between the lipid composition of plasma membrane and the CFTR protein [68]. It seems, therefore, that a correction of PL composition may improve CFTR activity *in vivo*, a hypothesis that calls for direct experimental validation in intestinal cells.

### II.3. Nutritional Status and Prognosis in CF

Various studies emphasize a correlation between a low nutritional status and an unfavorable prognosis in patients with CF [69]. Chronic undernutrition is associated with weight and height retardation in CF children. Early studies showed a correlation between the degree of malnutrition and the severity of pulmonary disease [69-71] and a slower rate of deterioration of pulmonary function was found in CF children without steatorrhea [72]. However, low values of EFA (linoleic acid and DHA) are not always correlated with anthropometric data or lung function [55]. CF survival and well-being are also correlated with malnutrition in CF [73]. This positive correlation between good nutritional status and long-term survival underscores the optimization of energy balance, nutrient intake, as well as intestinal absorption for CF patients.

Since EFA levels are abnormal in CF patients and EFAD may contribute to CF pathophysiology in animal models, it has been suggested that EFA supplementation has a beneficial effect in this disease. Nutritional studies demonstrate that oral and intravenous supplementations with n-3 FAs are efficient in increasing EPA and DHA incorporation in plasma, erythrocyte and platelet membrane. [56-58]. Oral supplementation with n-6 FAs increases plasma and erythrocyte membrane AA levels [22,74-76]. The clinical effects of EFA supplementation vary among studies: linoleic acid increases weight for height in CF children [19,22] and an improvement in pulmonary function is also observed with n-3 FA intake. Nevertheless, this valuable effect is not reported in all studies. [56-58] and the differences may be explained by the limited number of patients (under 20) and the short duration (between 4 and 6 weeks) that prevent the proper evaluation of any clinical benefit. Undoubtedly, further studies should focus on the relationship between dose recommendations, the degree of correction of plasma EFAs and clinical outcome. In particular, long-term lipid dosage adjustments are necessary to define the optimal n-3/n-6 ratio required in CF patients to prevent clinical manifestations.

In contrast to carbohydrates and proteins, lipids are poorly soluble in water. Their lipolysis into the aqueous phase of the intestinal lumen, as well as their transport into plasma, requires the formation of soluble complexes such as BAs and lipoproteins (LPs). Any deficiency or variation in these processes may impact on fat digestion and transport. Fat malabsorption in CF may result from the inefficiency of sequential events: the lipolysis of alimentary lipids, the effect of BAs on micellar solubilization, the integrity of intestinal mucus and enterocytes, multiple intracellular processes and LP secretion. These lipid digestive and absorptive steps will be discussed chronologically.

## III. Intraluminal Abnormalities

### III.1.1. Lipolytic Phase

TGs cannot be transported into the cell because of their size and their hydrophobic characteristics. The lipolysis process is required to produce smaller and more hydrophilic molecules, which can thus be absorbed. TGs can be hydrolyzed by two lipolytic enzymes in humans: gastric lipase and pancreatic lipase [77,78]. The enzyme postulated to be the main participant in gastrointestinal lipid digestion is pancreatic lipase. However, intra-gastric lipolysis accounts for about 20–30% of total gastrointestinal lipolysis *in vivo *and it has been suggested that it is increased in CF pancreatic insufficiency.

### III.1.1 Gastric Lipase

Human gastric lipase is the key acid enzyme that is secreted by the chief cells located in the fundic region of the stomach. Human gastric lipase is stable in gastric juice at pH values ranging from 2.0 to 7.0 and is the most stable acid lipase [79].

In the past, it was suggested that gastric lipase played only a minor role in the digestion of dietary fat, because of its specificity for medium-chain FAs, which are less common than long-chain FAs in dietary fat [80]. In fact, human gastric lipase also exerts an important activity on long-chain TGs in an acidic pH. When pH levels are between 5 and 6, it hydrolyzes medium-chain TGs more efficiently than longer ones [81]. This lipase is quantitatively important, contributing 20–30% of TG acyl chain hydrolysis [82-84]. Moreover, initial digestion of dietary fat in the stomach was found to be an essential step for optimal intestinal lipolysis for three reasons: releasing long-chain PUFA in the stomach induces cholecystokinine output, which stimulates pancreatic lipase secretion [85]; the efficiency of pancreatic lipase is increased with partially hydrolyzed TGs [86]; and free fatty acids (FFAs) enhance lipase binding to colipase [85].

Acid lipase activity exists in CF children [87,88], but its level, compared with normal controls, varies among studies [89,90]. Basal lipase activity in the stomach and in the duodenum is similar in CF patients and normal controls [91], but its lipolytic activity is almost three times higher in CF patients after a test meal [90]. There are two main explanations for the maintained high activity of acid lipase in CF patients. First, contrary to pancreatic lipase, the low postprandial pH in the upper small intestine, due to bicarbonate deficiency, does not inactivate gastric lipase activity [92,93] and the latter is preserved for a longer period than in normal subjects. Second, the inactivation of gastric lipase is less effective, because of the low concentration of BAs and pancreatic proteolytic enzymes [94,95]. Compensatory lipolytic activity by gastric lipase may account for 40–70% of dietary fat digestion [87,96]. In CF patients, preduodenal activity amounts to 90% of the total lipase activity at the ligament of Treitz in the postprandial state [96]. However, acid-resistant enzyme supplements, such as fungal lipase, fail to prove efficacy in CF [97]. No correlation between gastric lipase and EFA status is reported in the literature.

### III.1.2. Pancreatic Lipase and Colipase

Amphipathic molecules, monoacylglycerols (MG) and FA, produced during TG hydrolysis, stabilize dietary fat emulsion. Most of the fat digestion occurs in the upper small intestine following the action of pancreatic lipase and BAs. The hydrolysis of long-chain TGs is achieved by pancreatic lipase. Its optimum pH is 8 and the enzyme becomes inactive at a pH under 6 and is irreversibly inactivated at a pH less than 4.5. Colipase is required for pancreatic lipase stabilization on lipid droplets and for optimal lipolytic activity. At least, two other hydrolytic enzymes are needed to release FAs: PLA2 and cholesterol esterase (CET).

Fat malabsorption is present only when pancreatic enzyme secretion is under 10% of normal [98]. On diagnosis, only 15% of CF patients have sufficient pancreatic function for normal fat digestion [72]. The pancreatic function decreases with time, since 37% of CF newborns show a substantial preservation of pancreatic function [99] and more than 90% of CF infants and younger children display pancreatic insufficiency. The physiopathology of pancreatic insufficiency results from several abnormalities. In fact, pancreatic changes are caused by the obstruction of small ducts by thick, sticky secretions and cellular debris. A primary defect of fluid secretion (water and bicarbonate) leads to high protein concentration in pancreatic juice [100]. Then, protein precipitation blocks small pancreatic ducts and promotes pancreatic acinar atrophy and fibrosis. The ratio of acinar cells to connective tissue decreases progressively in the pancreas of CF children until the normal structure of the pancreas is lost. The role of the CFTR for normal proliferation and differentiation of secretory cell populations has been demonstrated in the lung and intestine of transgenic mice [101,102] and, interestingly, the CFTR appears as a valuable marker of human pancreatic duct cell development and differentiation [103]. CFTR mutation may, therefore, affect the proliferation of pancreatic secretory cells, thereby favoring pancreatic insufficiency and physiopathology.

Not only quantitative but also qualitative abnormalities characterize pancreatic lipase activity. Pancreatic lipase supplements are inactivated by acidic pH levels in the small bowel: only 8% of active enzyme reaches the ligament of Treitz [92,93]. The severity of pancreatic involvement appears to increase with age and, even with maximum pancreatic enzyme replacement therapy, a substantial proportion of ingested energy is probably malabsorbed.

There is little information about colipase in CF. Although, colipase has been reported to be deficient in this disease [104], its secretion is extremely variable in CF patients [105]. More investigations are necessary to clarify this aspect.

End products of gastric lipases are mainly one FFA and one 1-3-diacylglycerol, while the lipolytic products of pancreatic lipase are two FFAs (Sn-1 and Sn-3 postions) and one 2-monoacylglycerol [106]. Furthermore, in contrast to pancreatic lipase, gastric lipase has been shown to cleave not only the external ester bonds (n-1 and n-3 position), but also the ester bond in the Sn-2 position [107]. This difference is important for nutritional intervention with enteral defined structured TGs, which provide medium-chain FA as a source of immediate energy. In Caco-2 cell cultures, the addition of structured TGs containing only octanoic acid, a non-EFA, in the Sn-1 and Sn-3 position, but linoleic acid in the Sn-2 position in the presence of gastric lipase, enhances the cellular uptake of TGs, improves cellular EFAD and exhibits a beneficial effect on lipid incorporation and LP production [108]. In the rat, EFAs at the Sn-2 position appear faster in lymph than EFAs at the terminal positions of TGs [109] and are more readily recovered in lymph [110]. Defined TG with EFA linoleic acid at the Sn-2 position increases the absorption of EFA in rats with fat malabsorption induced by biliary and pancreatic diversion [111]. These results suggest that structured TG, in the presence of gastric lipase, could improve EFAD, despite the absence of pancreatic lipase activity. In patients who are not supplemented with pancreatic enzymes, an organized lipid matrix containing lysophosphatidylcholine (Lyso-PC), MG and FFA could be effective in increasing TG and retinyl-palmitate absorption and in improving clinical outcome [112].

In animal models, EFAD does not affect the lipolytic phase: there is no difference between EFAD and control rodents in the lipolytic step [113,114]. Furthermore, animal models suggest that EFAD is likely to be of minor importance in exocrine pancreas function in CF [113,115]. Furthermore, EFAD does not seem to induce any significant changes in the structure and composition of pancreas [116]. Overall, these studies show that structured TG can effectively supply functional FAs distributed throughout the glycerol skeleton. In particular, structured TG with EFAs at the Sn-2 position and medium chain FAs at the Sn-1 and Sn-2 positions of the glycerol molecule can be used for distinct nutritional and clinical purposes in CF. Although, it is obvious that structured TG are far superior to conventional oils, it is quite difficult to appreciate their influence on lipolysis in CF patients with EFAD in view of the paucity of information available in the literature. Probably, the difficulty originates from the enormous cost of producing highly purified structured TG.

### III.1.3. Phospholipase A2 (PLA2) and Cholesterol Esterase (CET)

The phospholipid-hydrolyzing enzymes include phospholipase A_1 _(PLA_1_), PLA_2 _and CET [117]. PLA_1 _and PLA_2 _are secreted in their zymogen form and activated by trypsin on entering the duodenum [118]. PLA_2 _preferentially hydrolyzes FA in phosphatidylcholine (PC), the most abundant dietary PL, at position 2 to produce more hydrosoluble components (FA and 2-lyso-PC). CET hydrolyzes CE, retinyl ester and lyso-PC in the small bowel [117], but it is 100 times less effective than pancreatic lipase in the TG digestion process.

In CF patients, PLA_1 _and PLA_2 _activity is correlated with the pancreatic function [119]. It is of particular interest to note that, despite the total absence of PLA_2_, around 30% of PLs are hydrolyzed [120]. The decreased activity of PL hydrolyzing enzymes may interact with lipid absorption in several ways. First, since PLs are amphipathic molecules, they adsorb to the surface of lipid droplets, preventing contact between the lipase-colipase complex and the TG substrate [118]. PC hydrolysis will allow desorption of lyso-PL from lipid droplets, which are relatively hydrosoluble molecules, and will thus facilitate the interaction between TG and pancreatic lipase. Secondly, unhydrolyzed PLs impair the absorption of cholesterol and FAs [121,122]. Finally, they impair bile acid absorption through their receptors in the terminal ileum, which increases bile acid losses in the stool.

In CF children, CET activity is dramatically decreased [123] and parallels pancreatic lipase activity. The reduction in BAs, which are essential cofactors for optimal esterase activity, results in a failure to solubilize and thus absorb cholesterol and their esters.

In the enterocyte, lyso-PC is an essential source of PLs for LP formation [124], the transport of TGs from the enterocyte into the blood [125] and the PL turnover induced by exocytosis [126]. These phenomena may be impaired by phospholipase or CET defects.

No data are provided about the impact of EFAD on PLA_1_, PLA_2 _and CET activity. However, AA and DHA decrease the secretion of bile-salt dependent lipase, but without directly impairing the biosynthesis of this enzyme. AA alters the transport of the enzyme toward the cytosol, leading to the retention of bile-salt dependent lipase in microsomes [127]. It is likely that much attention must be paid to phospholipases and CET in CF in view of their role in fat absorption. Future studies should be designed to test whether their supplementation may increase lipolysis efficiency and alleviate EFAD in CF.

### III.1.4. Intraluminal pH

Duodenum pH results from an interaction among food buffering, gastric acid production and pancreatic bicarbonate secretion. In humans, physiological pH is 2–4 in the stomach and it varies from 6–7 in the upper duodenum, but rarely dips below 5.

In CF patients, pancreatic, intestinal and biliary HCO3^- ^secretion is decreased to below 10% of the normal values [128]. Consequently, a low pH is observed in the intestinal lumen of the duodenum and even during postprandial time. Besides, basal and postprandial pH levels in the stomach are similar in CF patients and normal subjects [91,96]. In CFTR^(-/-) ^mouse intestine, HCO3^- ^secretion is impaired too [129,130]. The acidic upper small intestine in CF may contribute to fat malabsorption given: 1) the inactivation of trypsin, which induces an impairment of active enzyme molecules from their zymogen form (pancreatic lipase, PLA2); 2) the inactivation of pancreatic lipase [131]; 3) BA precipitation, decreasing the BA pool and diminishing its availability to form micelles; and 4) early protonation, which impairs the micellar dispersion of lipolytic products [132]. The ability of acid suppressant therapy (cimetidine, ranitidine or proton pump inhibitors) to improve fat absorption is controversial. Recently, a metanalysis of randomized trials involving agents that reduce gastric acidity has been carried out and it showed that treatment failed to improve nutritional status and had little impact on fat absorption [133]. Differences between studies could be due to the small number of subjects, differences in dietary intake and the degree of pancreatic insufficiency, the important physiologic range in fat intestinal absorption and variability in intestinal drug absorption or interindividual variation in medication bioavailability, pH measurement and fat absorption evaluation.

### III.2. Micellar Phase and Biliary Abnormalities

BAs are amphipathic molecules synthesized by the liver. They enhance the solubility of lipolytic products in the aqueous intestinal phase [134,135]. In fact, micellar solubilization increases the aqueous concentration of MG and FA 100 to 1000 times. The importance of this phase is underscored by a 30% reduction in dietary lipid absorption in patients with biliary atresia [136]. Similarly, rats with biliary drainage displayed reduced linoleic acid absorption [137].

#### III.2.1. BA Excretion

Outside the critical micellar concentration range, the amount of lipid solubilized is significantly reduced. Consequently, steatorrhea appears [138] and FA absorption decreases [139]. This situation is found in CF patients where total bile salt secretion is impaired [140]. About 36% of CF children showed reduced BA secretion into the duodenum [141]. Despite this low BA secretion, impaired water secretion leads to high concentrations of bile salts in hepatobiliary secretions [142], which may contribute to the cholelithiasis reported in CF patients [94,143].

EFAs regulate BA excretion: PUFAs are reported to induce the excretion of BA in rats [144] and humans [145], but this effect depends on the EFA family, since n-3 FA increases bile flow more than n-6 FA [146]. The EFAD effect on bile flow and BA secretion varies among animal species: decreasing in rats [147,148], having no effect in mice [114] and increasing in hamsters [114]. In humans, the impact of EFAD on BA secretion and composition remains unclear. However, prostaglandins, which are AA metabolites, alter hepatic bile flow [149] indicating that disturbances of AA status may affect choleretic response in CF patients.

#### III.2.2. BA Composition

The nature of BAs influences their ability to solubilize lipids. Cholesterol absorption by enterocytes is greater with cholyl-taurine than with chenodeoxycholyl-taurine [150-152] and BAs with conjugated taurine are more effective than glycine-conjugated BAs at solubilizing lipids. The effect of EFA on BA composition variation and fat absorption remains enigmatic in humans, since there are no available data.

In CF, oral taurine load appears to be normal, but excessive fecal loss [153] increases the ratio of glycine/taurine-conjugated BA [94,140,141]. Oral taurine supplementation in CF children is effective in decreasing the glycine/taurine ratio in duodenal fluid [153], but its ability to improve fat malabsorption is controversial [154-157]. Apparently, taurine supplementation improves BA malabsorption, mainly in patients with a high degree of steatorrhea [158-160].

Studies on animals show that EFAD leads to impaired biliary excretion of taurocholate [161] and to reduced EFA content (linoleic and arachidonic acid) in biliary PC that is essential for micelle formation [162]. However, BA and bile lipid composition vary across species. In rats and hamsters with EFAD, bile composition is markedly impaired [147,161]. On the other hand, BA composition appears to be similar in EFA-deficient and EFA-sufficient mice [114].

The relationship between BA and EFA was investigated in CF-EFAD children and it showed that ursodeoxycholic acid supplementation improves the hepatic metabolism of EFA. After 1 year, ursodeoxycholic acid supplementation led to an improvement of EFA status (reduction of triene/tetraene FA ratio) [163].

#### III.2.3. BA Malabsorption

BAs are recycled through the enterohepatic cycle with remarkable efficiency (95% reabsorbed) [164,165]. The enterohepatic cycle is often interrupted in CF because of excessive fecal losses of BA, [95,166-170], which diminish the BA pool [171,172]. Excessive BA losses may be attributed to: 1) BA irreversibly bound to unhydrolyzed TG and PL within the intestinal lumen [173] ; 2) BA precipitation due to acidic pH in the duodenum [174]; and 3) intestinal bacterial overgrowth, which is present in 40% of CF patients and results in BA deconjugation and dehydratation [175]. BA loss may also result from a primary cell defect in the active absorption of BA in the ileum. *In vitro *studies using brush border membrane vesicles from CF patients have shown that total ileal BA uptake is diminished [176,177]. Surprisingly, a study of Ileal Biliary Acid Transporter (IBAT) in CFTR knock-out mice shows a BA uptake rate four-fold that of wild-type mice [178]. The increase in IBAT protein and BA uptake can be interpreted as an up-regulation in response to a low BA rate. This result should lead to a renewed interest in intraluminal events, especially for the implication of the thick mucus barrier in CF pathophysiology.

#### III.3. Gastric and Intestinal Transit Time

Fat digestion begins in the stomach with the action of acid lipase. Following hydrolysis by gastric lipase, medium-chain FAs are partly absorbed by the stomach [179]. Thus, the stomach plays an essential role in fat digestion, especially in pancreatic insufficiency [147].

In CF patients with pancreatic insufficiency, altered motility with an increase in gastric emptying [180] and small bowel transit time has been described [181-184]. It has been reported that slow gastric emptying reduces the success of pancreatic enzyme replacement therapy in improving TG hydrolysis, which could explain in part the variation in pancreatic enzyme replacement therapy efficiency [180,185]. However, other studies have shown that gastric emptying time is similar in both CF patients and healthy controls [90,182].

In EFAD rats, AA and linoleic acid are emptied from the stomach at similar rates and these rates do not differ from controls [186]. However, EFA-deficient mice show that the motility of epithelial cells is increased in the jejunum [187].

### III.4. Intestinal Mucosa Trophicity

#### III.4.1. Quantitative and Qualitative Mucosal Abnormalities

In the majority of CF patients, histological brush border studies reveal normal morphology [188]. However, in some cases, abnormalities are reported, such as ileal hypertrophy [189] or partial villous atrophy in the small intestine [190,191]. This atrophic mucosa, which also occurs in knock-out mice [189], may result from acidic aggression by unbuffered stomach chyme, chronic inflammation or denutrition. Furthermore, a thick mucinous layer covering the brush border and large areas of the microvilli is revealed by electron microscopic examination of biopsies from CF patients [188], which may contribute to malabsorption in CF patients. The viscosity of the intact CF glycoprotein is almost two-fold that of normal glycoprotein [192]. Elevated viscosity may be caused by defective chloride transport, mucin hyperglycosylation or a high level of disulfide-linked peptides [193,194]. Obviously, the highly acidic properties of surface components related to oversulfatation could modify the micro acidic climate on the intestinal epithelium surface and influence interaction between micelles and enterocytes, impairing the protonation of FAs.

In EFAD, histological and biochemical alterations of the intestinal mucosa are described [195]. In rats and mice with EFAD, the height of villi is decreased leading to a diminished absorption area. Cellular differentiation was also found to be impaired [196], highlighting the role of EFA in the formation of new tissues, such as the maintenance of tissue and cell structure [197]. Furthermore, the abnormal structure of mitochondria and microvilli is correlated to decreased fat absorption [196]. It seems difficult to reconcile jejunal hypertrophy in CF patients and microvilli in EFAD animals. However, EFAD seems to induce histological abnormalities in the CFTR function. In CFTR knock-out mice, jejunal hypertrophy is corrected with an oral administration of high doses of DHA [198], which are associated with an inhibitory proliferative cell effect [199]. Then, more than in typical EFAD, an abnormal EFA imbalance could lead to hypertrophic villosities in CF patients. Hypertrophy is only localized in the jejunal portion. Accordingly, the FA composition of jejunum mucosa, the main segment for optimal lipid absorption, is markedly different from ileal and colonic mucosa [195]. Furthermore, it is interesting to note that dietary influences are tissue specific, since serum or red blood cell membranes do not reflect local changes in any of the different intestinal segments [195]. At present, no studies have correlated intestinal morphology in CF patients with their EFAD status.

#### III.4.2. Intestinal Permeability

Recently, studies have suggested that pathological modifications in EFAD may be the consequence of cell adhesion disorders [200].

In CF patients, the dual sugar permeability test [201] demonstrates that the paracellular pathway is more permeable to large molecules, while the passive transcellular uptake of small molecules is normal [182]. Intestinal permeability in CF is related to patient genotype: patients homozygous or heterozygous for ΔF508 mutation exhibit significantly increased intestinal permeability compared with patients with unidentified genotypes or controls [202]. Moreover, abnormalities in the tight junction, an essential structure for the control of intestinal permeability, have been reported in the intestinal epithelium of fetuses with CF [203].

EFAs are able to modify cells ultrastructurally and to alter intestinal permeability. In the culture of enterocytes from EFA-deficient CF, the lateral surfaces between cells are fairly straight, a consequence of the absence of complex interdigitations that play an essential intercellular cohesive role [196]. Moreover, a reduction in the number of desmosomes has been reported in the intestinal tract of EFAD rats [59,204]. Studies in endothelial cells suggest a possible mechanism of EFA-modulating cell adhesion. However, this EFA function has to be demonstrated in intestinal cells. In endothelial cells, some PUFAs such as γ-linolenic acid or EPA increase transepithelial electrical resistance and reduce paracellular permeability. Studies involving CF patients are definitely needed to assay intestinal cell adhesion molecules and their relation to FA status.

## IV. Enterocyte Phase Abnormalities

### IV.1. Enterocyte Lipid Uptake

#### IV.1.1. Physiology

EFAs may be absorbed by enterocytes, mostly in the form of EFA and MG. Until recently, it was assumed that these lipids diffused passively through the enterocyte brush border membrane [205]. Indeed, earlier studies reported that the uptake of FAs occurred at 0°C, implying that the process is strictly passive. In particular, the intestinal absorption mechanism of linoleate was noted to depend on its intraluminal concentration, showing a passive diffusion at high concentrations. However, a transporter was required at weaker concentrations [206]. Accordingly, the absorption of FFA was found to be a saturable phenomenon that can be inhibited through competition with long-chain PUFA [207]. These observations suggest that FA uptake is a concentration-dependent dual transport mechanism involving both passive diffusion and a carrier-dependent process. Recently, several membrane transport systems have been identified and they seem to be involved in the enterocyte absorption of lipids: membrane FA binding protein (FABPm), capable of facilitating transmembrane passage mainly of FAs, but other lipids as well [208-210]; FA translocase/cluster determinant 36 (FAT/CD36) implicated in long-chain FA transport. [211-213]; scavenger Receptor class B type I (SR-BI) that plays a role especially in cholesterol movement (absorption and/or efflux) at the enterocyte level [214]; ATP Binding Cassette transporter family that provides several cholesterol carriers: ABCG5 and ABCG8 cooperate to limit sterol intestinal absorption, rather facilitating cholesterol efflux toward the intestinal lumen and their mutations predispose to sitostrolemia [215,216]; and ABCA1 expressed in the enterocyte, which could partially control cholesterol efflux toward the intestinal lumen [217], although its exact role in the brush border membrane remains controversial [218,219]. Recently, a new protein called Nieman Pick C1-Like1 was identified in the small intestine. It seems closely involved in intestinal cholesterol absorption, a pathway sensitive to sterol absorption inhibitors such as ezetimibe [220,221].

#### IV.1.2. CFTR and Lipid Transporters

No study to our knowledge has investigated the interaction that may exist between the CFTR and these lipid transporters. Yet, the CFTR is known to modulate the activity of other carriers as well as certain ionic channels, for instance [37,222]. The CFTR regulation of other intestinal ionic transporters is effectively diminished in CF patients [223]. Furthermore, a microarray study on pulmonary tissue from knock-out CFTR mice shows that membrane transporters specific for ligands as different as glutamate, hormones or neurotransmitters have their expression influenced by CFTR [224]. The completion of the same type of study on intestinal tissue would likely offer interesting tracks to target lipid transport proteins capable of being influenced by the CFTR. The large diversity of transporters interacting with the CFTR could lead to impairment in enterocyte lipid uptake and trafficking in CF, which would represent another cause for nutrient malabsorption.

The existence of anomalies in the enterocyte uptake to EFA is controversial. In effect, EFA intestinal absorption in patients does not always appear to be impaired. Some studies report that the rate of linoleic acid absorption is normal when pancreatic enzyme supplementation is given at sufficient doses [225]. Apparently, even the presence of steatorrhea was not accompanied by diminished EFA absorption [186] and no correlation has been established between steatorrhea and EFAD in preadolescents with CF [226]. On the other hand, a recent study has shown that patients undergoing pancreatic enzyme treatment display a reduction in the intestinal uptake of long-chain FAs [9]. The differences between these studies may indicate either that the intestinal malabsorption does not in itself explain the EFA deficit or that the severity of CFTR mutation could influence the enterocyte absorption of lipids. Unfortunately, genotyping analysis has not been carried out in most of these investigations. Future developments will get to the bottom of these major unsolved questions by tracing the defects in enterocyte lipid uptake, the status of lipid transporters, the relationship with CFTR in CF patients and fat malabsorption.

#### IV.1.3. EFAD and Lipid Transporters

Early studies on EFAD did not notice an anomaly in the enterocyte transport of EFA [186,196,227]. However, these data have not been confirmed. The studies are somewhat outdated and the sensitivity of the techniques used may be called into question. Furthermore, several elements suggest that EFAD may be implicated, at least to some extent, in lipid intestinal malabsorption. Indeed, EFAD can affect the lipid composition of the enterocyte membrane and modify its fluidity, which may directly disturb the functioning of the transporters that can be found there. Moreover, some transporters like the SR-BI or CD36 could act as transporters, not directly, but by creating a special micro-environment in the neighbouring membrane lipids. This local change favors the transfer of their ligands [228]. In this model, it appears very likely that a modification of the physiochemical properties of the membrane, as is the case in EFAD, may impact on the transporter lipid transfer abilities. EFAD could also influence these lipid transporters more directly. In effect, most of these transporters are regulated by long-chain FAs: the expression of the FABP and CD36 genes is increased by long-chain FAs [229]; the SR-BI protein is also regulated according to the type of long-chain FAs (unpublished personal data); and finally, the polyunsaturated FAs trigger a decrease in the ABCA1 protein, thereby reducing the basolateral efflux of cholesterol in human Caco-2 cells [230]. This regulation could be drilled through a reduction in ABCA1 expression [231] or an increase in the degradation of the protein [232].

FAs can also act directly on the level of expression of the CFTR protein. In this way, a short-chain FA, the butyrate, increases CFTR expression significantly in animal epithelium cell cultures [233]. It is interesting to note that this same FA modulates lipid synthesis, the biogenesis of apolipoproteins (apos) and the assembly of LP in the enterocyte [234]. To our knowledge, there are no similar studies with long-chain FAs or EFAs. The potential role for the CFTR in all the enterocyte lipid synthesis steps underlines the need for new investigations, which may lead to new therapeutic strategies.

### IV.2. Enterocyte Lipid Trafficking

#### IV.2.1. Physiology

After crossing the brush border membrane, the lipids must be processed by cytosolic proteins for their intracellular trafficking toward various compartments, including the endoplasmic reticulum (ER), where their reesterification may take place. However, the precise mechanisms behind this transport remain unclear. Certain transport proteins have been identified, but their roles often remain hypothetical: the Sterol Carrier Protein (SCP-2) and two cytosolic FABP capable of linking FA in particular, but also PL. According to our unpublished and preliminary data, intestinal-FABP (I-FABP), which is restricted to the intestine, could direct the FAs to membranes for lipid cycling or to a degradation pathway (peroxisome or mitochondria), whereas the liver-FABP (L-FABP), also found in the liver, could guide them to the ER to be assembled into LP. I-FABP and L-FABP bind differently according to lipid class, but both with a greater affinity for unsaturated FAs than saturated FAs.

#### IV.2.2. CFTR and Enterocyte Lipid Trafficking

No study has examined the interactions that can exist between the various proteins of the cytoplasmic transport of lipids and the CFTR. However, it has been clearly demonstrated that anomalies in CF exist in the intracellular movement of the CFTR itself. Various physiochemical conditions, such as low temperatures [233] or chemical agents [235], are able to increase the stability of the mutated CFTR protein and avoid its abnormal retention in the ER [236,237]. The latter would, in part, be attributable to a defective interaction between the CFTR and certain PLs that play the role of lipid chaperones. Obviously, the mutation of ΔF508 causes the CFTR protein to lose its ability to bind preferentially with phosphatidylserine rather than with PC. The replacement of PC with non charged analogues in mutated cell cultures increases CFTR expression and the quantity of its mature form. Therefore, certain PLs through their lipid chaperone function seem important for the intracellular trafficking of the CFTR [238]. Moreover, the invalidation of the CFTR initiates changes in gene expression as well as protein degradation via ubiquitin-dependent proteasome, which may modify several transport proteins [224]. Overall, these studies support the concept that CFTR is modulated by PLs and indicate potential relationships between CFTR and other local transporters. Unfortunately, it remains unknown how EFAs contained in PLs directly alter the activity of CFTR and whether a "partnership" exists between CFTR and intracellular lipid transporters in the enterocyte. Forthcoming investigations will highlight new links between the CFTR, EFAs and cellular processes, which may identify important factors that play a role in networks of lipid signalling and transport.

#### IV.2.3. EFA and Enterocyte Lipid Trafficking

The assembly of microtubules is critical for intracellular trafficking and chylomicron transport [239,240]. Early reports had indicated that the administration of microtubule inhibitors led to a decrease in the conveyance of radioactive lipids in rat enterocytes [241]. Certain EFAs, such as γ-linoleic acid or AA, regulate the microtubule polymerization [242,243]. Thus, EFAD could hinder intraenterocyte trafficking through an alteration of microtubule polymerization, synthesis or function.

### IV.3. Lipid Esterification and Lipoprotein Synthesis

#### IV.3.1. Physiology

The products of lipolysis once absorbed and transported to the ER are resesterified to form TG, PL and CE through specific enzymatic pathways. This reesterification involves several enzymes monoacylglycerol acyltransferase (MGAT), diacylglycerol acyltransferase (DGAT), glycerophosphate acyltransferase, phosphatidate phosphodiesterase, lyso-PC acyltransferase, CE and acyl-coenzyme A:cholesterol acyltransferase (ACAT). After their synthesis, hydrophobic lipids must be associated with proteins or apolipoproteins, in order to allow their solubilization in the blood circulation, thereby forming complexes known as LP. The intestine is capable of secreting most lipoprotein classes (chylomicrons, VLDL, HDL), but chylomicrons represent the specific and most abundant class in the enterocyte.

Note that apo B is a component that is essential for LP assembly and secretion by the intestine. It exists in two forms: apo B-100, present particularly in the liver, but to a limited extent in the intestine, and apo B-48, which is specific to the enterocyte that results from the posttranscriptional modification of the apo B-100 mRNA, or editing, involving an enzymatic complex called APOBEC-I (apo B mRNA-editing catalytic subunit-1) [244]. Other apos are produced in the enterocyte, mainly apo A-I and apo A-IV that is exclusively of intestinal origin in humans [245]. During its synthesis in the ER, apo B must undergo lipidation that protects it from degradation by the proteasome [246]. Lipid transfer from the endoplasmic reticulum to apo B requires microsomal triglyceride transfer protein (MTP) intervention. This step is crucial for chylomicron assembly as noted in abetalipoproteinemia, an illness brought on by the mutation of the MTP gene, where there is defective lipoprotein secretion [247].

Pre-chylomicrons are exported to the Golgi apparatus where they undergo their final maturation (glycosylation of the apos, modification of certain PLs, etc.) before being secreted through the basolateral membrane into the lymphatic milieu. It is important to note the role of cargo proteins in the secretion step, which has been highlighted in chylomicron retention disease: the mutation of the Sar-1 GTPase protein prevents the secretion of chylomicrons through the dependent COPII vesicles [248]. TG transfer from the ER to the Golgi apparatus seems to be a limiting step in fat absorption [249]. After being secreted into the lymphatic capillaries, intestinal LPs are discharged into the systemic blood circulation through the thoracic canal.

#### IV.3.2. Role of the CFTR in the Intracellular Phase

In CF patients, lipid composition, concentration and size are irregular [250]. At present, the role played by CFTR anomalies is unknown. To our knowledge, no study has focused on the possible interactions of the CFTR with the implicated enzymes in lipid reesterification, apo B biosynthesis, MTP activity or the relationship with Sar-1 GTPase protein expression.

It is during this LP secretion step that interactions with the CFTR may be the most likely. In fact, CFTR mutations alter the secretion not only of electrolytes, but also of substances as different as BAs by the hepatocyte [172], γ-light chain antibodies by lymphocytes [251], INFγ by monocytes [252] or neurotransmitters by pulmonary neuroendocrine cells [253]. Since the enterocyte represents a key cell in the physiopathology of CF and given the numerous lipid aberrations observed in this disease, it appears essential to study the secretion abilities of lipids by epithelial cells instead of focusing only on the digestive mechanism in CF as is presently the case. For example, the recent analysis of RNA, influenced by CFTR knock-out, identified a number of proteins involved in LP metabolism, including proteasome 26S [224]. The latter subunit is the major proteolytic component of the ubiquitin-dependent proteasome. As mentioned before, the ER-localized ubiquitin-proteasome pathway is primarily involved in the intracellular degradation of apo B. Its alteration in the absence of CFTR [224] indicates potential relationships between CFTR and the apo B recovery/degradation pathway, thereby determining LP assembly and secretion. Furthermore, there is increasing evidence that CFTR regulates endosomal fusion and vesicular trafficking [254], indicating potential relationships between the CFTR and ADP-ribosylation factor [254], which has a central role in VLDL assembly [255]. Finally, the Sar-1/COPII complex is necessary when apo B-100 exits the hepatic ER [256] and apo B-48 containing chylomicrons are exported from the enterocyte [248]. However, the Sar-1/COPII complex is also implicated in the exiting of CFTR from the ER [257] for its entry into the proteasome degradation pathway [258]. Overall, these observations demonstrate the existence of possible interactions among apo B elaboration, chylomicron packaging and CFTR function. Hence, CFTR mutations may significantly affect lipid transport and obviously studies are needed to clarify this relationship.

#### IV.3.3. Possible Implications of EFAs in Intracellular Lipid Transport

It is important to underline that the anomalies in the reported LP during CF are most marked in patients with EFAD [250]. These defects may either be the direct consequence of EFAD on LP synthesis or may only reflect an association between the type of CFTR mutation and lipid metabolism. A phenotypic classification of LP profiles according to genotype would allow us to elucidate this question. In fact, EFAD could cause interference at several levels within the process of LP synthesis and secretion.

TG reesterification rates are diminished in rats during EFAD [259,260]. Lipid membrane modifications, notably long-chain FAs, are known to change the functioning of reesterification key enzymes such as MGAT [261]. Similarly, intake rich in (n-3) FA lowers the ratio (n-6)/(n-3) and leads to an activation of DGAT and ACAT [262]. Therefore, an unbalancing of the EFA status could affect the lipid reesterification step.

Biosynthesis through the enterocytes of several apos, such as apo B or apo A-IV, is regulated in a specific way by certain EFAs [263-265]. Experimental studies confirm investigations carried out on humans [266]. Thus, EFAD is possibly deleterious for the synthesis of the main intestinal LP. Accordingly, enterocyte secretion of synthesized lipids is impaired during EFAD in mice and rats, which translates into an accumulation of large lipid droplets in the intercellular space [196]. Notably, in EFAD, the balance between the various EFAs rather than directly the absence of EFAs could impair the exocytosis mechanism. In effect, in enterocyte cultures, certain EFAs, such as EPA, decrease TG esterification and PL transfer from the ER to the Golgi and the mechanisms responsible for these processes have not yet been specified [267]. Similarly, EFAD may affect microtubules [242-268], as we previously mentioned, which could impact on the assembly and secretion of chylomicrons [239,240,242].

## V. Conclusion

It is well established that defective digestive processes in CF patients are secondary to pancreatic insufficiency. However, it is difficult to reconcile the failure of appropriate pancreatic enzyme replacement therapy with the persistent fat malabsorption. Since mutations in CFTR result in impaired intracellular pH organelle, glycosylation and sialylation in mammalian cells, it is possible that disturbances in intestinal CF lipid transport may also be associated with cause-related changes in the second step of fat absorption, i.e. the intracellular phase leading to lipolytic product uptake and esterification, apolipoprotein synthesis and processing, and nascent lipoprotein assembly and secretion following the fusion of Golgi vesicles with the basolateral plasma membrane. This hypothesis is reinforced by the findings that CFTR dysfunction alters further intracellular pathways, crucial for lipoprotein packaging and delivery, such as ubiquitin-proteasome complexes, endosomal fusion and vesicular trafficking, as well as Sar1/COP II and ADP-ribosylation factor 1/COP I systems. Additionally, in keeping with substantial growing evidence from the available literature, it is reasonable to put forward that EFAD contributes to CF malabsorption by interfering with intra-enterocyte lipid transport. If previous studies had entirely attributed EFAD to diminished EFA intake and malabsorption, it is likely today that a mutated CFTR may also decrease the incorporation of EFAs in PL [42], increases the release of AA [49], lowers the concentrations of linoleic acid and DHA, and disrupts EFA metabolism [42,50]. An interrelation between CFTR and EFAs is demonstrated when the chloride channels are blocked chemically [41]. Nevertheless, the exact relationship between CFTR and EFAs remains unclear and studies in this fundamental direction would shed considerable light on our understanding of the mechanisms responsible for EFAD in CF. This aspect is particularly important given the suggestions that EFAD is related to a basic defect in FA metabolism. It seems imperative that rigorous and long-term studies be conducted on EFA supplementation to normalize EFA status in CF patients, otherwise EFAD would continue to affect intestinal lipid transport and to simultaneously exacerbate the poor clinical course of the CF patients.

## Abbreviations

AA (20:4n-6): Arachidonic Acid

ABCA1: ATP Binding Cassette transporter A1

ACAT: Acyl-coenzyme A:cholesterol acyltransferase

Apo: Apolipoprotein

BA: Biliary Acid

CD36: Cluster Determinant 36

CE: Cholesterol-Ester

CET: Cholesterol esterase

CF: Cystic Fibrosis

CFTR: Cystic Fibrosis Transmembrane Conductance Regulator

DGAT: Diacylglycerol acyltransferase

DHA (22:6n-3): Docosahexaenoic Acid

EFA: Essential Fatty Acid

EFAD: Essential Fatty Acid Deficiency

EPA (20:5n-3): Eicosapentaenoic acid

ER: Endoplasmic Reticulum

FA: Fatty Acid

FABP: Fatty Acid Binding Protein

FFA: Free Fatty Acids

IBAT: Ileal Biliary Acid Transporter

LP: Lipoprotein

Lyso-PC: Lyso-Phosphatidylcholine

MG: Monoacylglycerol

MGAT: Monoacylglycerol Acyltransferase

MTP: Microsomal Triglyceride Transfer Protein

PC: Phosphatidylcholine (lecithin)

PL: Phospholipid

PLA2: Phospholipase A_2_

PUFA: Polyunsaturated Fatty Acid

SR-BI: Scavenger Receptor class B type I

TG: Triacylglycerol

## Competing interests

The author(s) declare that they have no competing interests.
